# Xin Su Ning—A Review of Basic and Clinical Pharmacology Integrated With Traditional Chinese Medicine Antiarrhythmic Theory

**DOI:** 10.3389/fphar.2021.657484

**Published:** 2021-11-11

**Authors:** Xuan Wang, Taiyi Wang, Shuwen Ding, Yu-Ling Ma

**Affiliations:** ^1^ The Oxford Chinese Medicine Research Centre, MSD, University of Oxford, Oxford, United Kingdom; ^2^ Shandong University of Traditional Chinese Medicine, Jinan, China

**Keywords:** antiarrhythmia, TCM theory, cellular electrophysiology, randomized clinical trial, pharmacology, Xin Su Ning, multicomponent medicine

## Abstract

Xin Su Ning (XSN) is a patented multicomponent medicine, which was certified in 2005 by the China State Food and Drug Administration to be produced pharmaceutically and to be used clinically. The XSN capsule was developed from an effective formula composed by Prof. Shuwen Ding of Shandong University of Traditional Chinese Medicine. Through more than 30 years of clinical observation, Prof. Ding concluded that XSN has a significant effect on arrhythmia with phlegm-heat heart-disturbed syndrome according to the traditional Chinese medicine (TCM) diagnosis. XSN, derived from a classical TCM formula Huanglian Wen Dan Decoction, is formulated with 11 Chinese herbal medicines to treat cardiac ventricular arrhythmia. Clinical evidence suggests that it is particularly efficacious for the arrhythmias induced by cardiac ischemia and viral myocarditis without obvious adverse reactions being reported. Cellular electrophysiological studies in ventricular myocytes revealed that XSN prolongs the duration and suppresses the amplitude of the action potential (AP), which is supported by the blockage of sodium and potassium channels indicating the characteristics of class I and III antiarrhythmic drugs. A recently reported double-blind, placebo-controlled, multicenter clinical trial of XSN enrolled 861 patients (ChiCTR-TRC-14004180) and showed that XSN significantly inhibited premature ventricular contraction (PVC). The cellular electrophysiological discoveries provided the mechanistic evidence for the clinical efficacy on inhibition of PVC by XSN as demonstrated in the clinical trial. These studies, for the first time, provided exclusive evidence that multicomponent TCM antiarrhythmic medicine can be evaluated using conventional research methods that have been used for antiarrhythmic drug discoveries for decades. We aimed to give a comprehensive review on XSN including its origin with the support of TCM theory, its pre-licensing clinical use and development, and its pharmacological and clinical study discoveries. The review will be summarized with the discoveries reported in a novel network pharmacological study that introduced a weight coefficient, which made it possible to evaluate the pharmacological properties of the TCM formula with regard to its formation based on TCM theory. Limitations regarding XSN’s basic and clinical research and possible future studies are listed. We hope that the advances in how XSN was studied may offer useful guidance on how other TCM could be studied with respect to the integrity of the TCM formulas.

## 1 Introduction

Chinese medicine has been practiced and developed for thousands of years along with the Chinese civilization. It is one of the well-developed traditional medicines in the world with comprehensive theories to guide the everyday practices; traditional Chinese medicine (TCM) accumulated rich medical knowledge in treating patients with sufficient efficacies. A distinguished feature of TCM is its multi-herbal, hence multicomponent nature, which is designed to act systematically to restore the tilted balance of the body evidenced with various diseases. The wide range of theories guiding TCM including holistic considerations, syndrome differentiation and treatment, and yin–yang theories are fundamental in deriving the most effective treatments.

Cardiac arrhythmia (arrhythmia) refers to the abnormal frequency, rhythm, origin, and conduction velocity or excitation order of the heart impulses. Arrhythmia is a common cardiovascular disease, which has serious health consequences. Arrhythmia can be caused by many cardiovascular diseases such as coronary heart disease, rheumatic heart disease, hypertensive heart disease, and viral myocarditis, and it can also be caused by the side effects of drugs. Treating arrhythmia with currently prescribed drugs or radiofrequency ablation all have limitations, particularly in elderly patients, who have increased risks of hypertension, diabetes mellitus, and coronary heart diseases. In addition, many antiarrhythmic drugs may inhibit certain cardiac functions and actually cause further arrhythmia, or the proarrhythmic effect, as side effects, which limits the clinical use of existing drug treatments due to poor tolerance and compliance (Huang and Wang, 2004; Li, 2011).

Amiodarone and other class I and III antiarrhythmic drugs are commonly prescribed to treat arrhythmia. Amiodarone, whilst widely considered effective in treating supraventricular and ventricular tachyarrhythmia, presents significant side effects. Chronic use of amiodarone causes serious adverse effects to several organs and tissue types, including the heart. Cardiac adverse reactions may include impairment to sinus beat formation and conduction and inducing significant bradycardia, with disproportionate impact on patients with pre-existing conditions, leading to dangerous polymorphic ventricular arrhythmia or torsade de pointes (Colunga Biancatelli et al., 2019). Amongst other side effects, ocular alterations are the most frequent with up to 98% of patients experiencing corneal microdeposits. The adverse reactions of other class I and III antiarrhythmic drugs are also well studied and documented due to decades of use (Caron and Libersa, 1997; Ji et al., 2017). These illustrate a clear demand for effective drugs with better safety profiles for chronic use.

Treating arrhythmia using TCM may have potential advantages. The XSN capsule is derived from an effective formula composed and used by Prof. Shuwen Ding of Shandong University of Traditional Chinese Medicine, which was built on decades of accumulation of knowledge and practical experience in treating arrhythmia. Prior to approval by a regulatory authority, XSN was used as a physician-prescribed decoction by Prof. Ding for over 3 decades, and it was certified in 2005 by the China State Food and Drug Administration to be produced pharmaceutically and to be used clinically. XSN is the first TCM approved for the treatment of arrhythmia with specific characteristics according to Chinese medicine theories, which were termed phlegm-heat heart-disturbed (PHHD) syndrome. XSN received National New Medicine Certification (Z20050131) and has been included in the Chinese Pharmacopoeia (2015 Edition). Based on the pathogenesis of PHHD syndrome arrhythmia (tachyarrhythmia), XSN was developed from Huanglian Wen Dan Decoction recorded in “Liu Yin Tiao Bian” written by Tingzhen Lu in the Qing dynasty, with the addition of Qinghao and Changshan, which are known for their anti-malarial and anti-arrhythmia properties (Ding et al., 2018).

The formula of XSN is composed of the herbs in proportion as follows: 300–360g Coptidis Rhizoma (Huanglian, *Coptis chinensis* Franch.), 225–265 g Pinelliae Rhizoma (Banxia, *Pinellia ternata* [Thunb.] Makino), 225–265 g Poria (Fuling, *Poria cocos* [Schw.] Wolf), 150–180 g Aurantii Fructus Immaturus (Zhishi, *Citrus aurantium* L.), 225–265 g Dichroae Radix (Changshan, *Dichroa febrifuga* Lour.), 438-50 g Nelumbinis Plumula (Lianzixin, *Nelumbo nucifera* Gaertn.), 225–265 g Sophorae flavescentis Radix (Kushen, *Sophora flavescens* Ait.), 225–265 g Artemisiae annuae Herba (Qinghao, *Artemisia annua* L.), 150–180 g Ginseng Radix et Rhizoma (Renshen, *Panax ginseng* C. A. Mey.), 225–265 g Ophiopogonis Radix (Maidong, *Ophiopogon japonicus* (L.f) Ker Gawl.), and 150–180 g Nardostachyos Radix et Rhizoma (Gancao, *Glycyrrhiza uralensis* Fisch.) (Wang, 2005).

### 1.1 Aim of the Review

We aim to present a comprehensive review of XSN that would answer the following questions:

1) The development history of XSN as an antiarrhythmic TCM
2) What are the pharmacological properties of XSN as a multicomponent antiarrhythmic medicine?
3) Why does XSN have to be multi-herbal and formulated with the 11 particular herbs?
4) What are the TCM theories that guide the formation of the XSN formula?
5) What are the pharmacological properties of the individual herbs that formulated XSN?
6) What are the pharmacological properties of some of the compounds isolated from these herbs?
7) Why does XSN not display proarrhythmic reactions while effectively treating cardiac arrythmias?
8) The limitation of the available basic and clinical studies of XSN and possible further studies

## 2 Pharmacological and Toxicological Studies of Xin Su Ning

### 2.1 Pre-Licensing Pharmacodynamic Studies

These data were filed by the China State Food and Drug Administration for approval of production and clinical use of XSN in China (Wang, 2005).

The pre-licensing pharmacological studies showed that XSN significantly suppressed cardiac arrhythmia induced by the chemical reagents matrin, calcium chloride, chloroform, and isoproterenol. In cardiac ischemia–induced arrhythmia, XSN can delay the onset of ventricular arrhythmia and shorten the time of arrhythmia. XSN could also lower the total cholesterol level of normal rats and reduce blood viscosity, hematocrit value, and fibrinogen.

1) Improve hemorheology

The high (2.56 g/kg/d), medium (1.03 g/kg/d), and low (0.51 g/kg/d) dose of XSN can significantly reduce the blood lipid of rats and can improve the hemorheology index of rats (*p* < 0.01).

2) Anti-ischemia–induced arrhythmia

The high, medium, and low dose groups of XSN can significantly reduce the incidence of Q wave, VP, and VT induced by coronary artery ligation (*p* < 0.05).

In addition, XSN can also prolong the incubation period of arrhythmia (*p* < 0.05) and shorten the duration of arrhythmia (*p* < 0.05), suggesting that it has an obvious inhibition effect on arrhythmia induced by coronary artery ligation in rats.

3) Protect the heart from I/R-induced damage

High, medium, and low dose groups of XSN significantly prolonged the incubation period of arrhythmia induced by myocardial ischemia-reperfusion (I/R) (*p* < 0.05), shortened the duration of arrhythmia (*p* < 0.05), reduced the incidence and mortality of VT and VF, and reduced the degree of ST elevation (*p* < 0.05).

### 2.2 Pre-Registration Toxicological Studies

1) Acute toxicity test

The maximum dosage of XSN was 43.74 g/kg/d in mice, which was about 500 times the daily dosage of clinical adults (Wang, 2005). Histopathological examination showed no pathological changes related to administration in the heart, liver, spleen, lung, kidney, and adrenal gland. No abnormal changes in weight, hematology, and blood biochemical indexes were found in the recovery period.

2) Long-term toxicity test

The long-term toxicity test was carried out in rats for 90 days, followed by recovery for 20 days. The dose administrated was 5.25 g/kg/d, which was about 60 times the daily dosage for clinical adults. Histopathological examination in the heart, liver, spleen, lung, kidney, and adrenal gland showed no pathological changes related to the long-term administration of XSN. No abnormal changes in weight, hematology, and blood biochemical indexes were found in the recovery period.

### 2.3 The Cellular Electrophysiological Properties of Xin Su Ning

**FIGURE 1:**
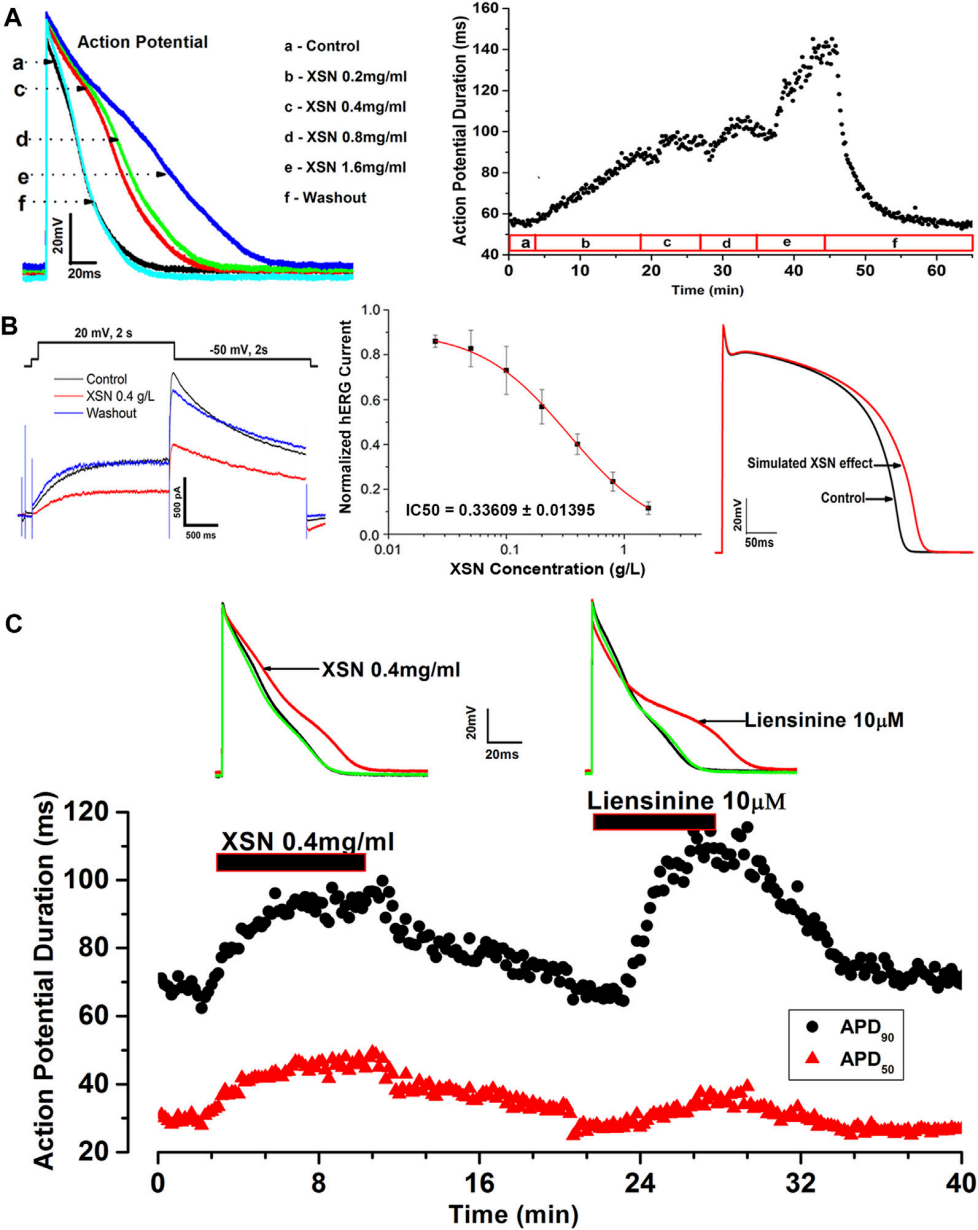
Effect of XSN and liensinine on APD of cardiac myocytes and computational simulation of human AP. **(A)** Data plot shows the concentration-dependent and reversible effect of XSN on APD, from which the superimposed AP traces were extracted to show the effect of XSN at various concentrations indicated by the keys. **(B)** XSN reversibly blocks the hERG channel in a dose-dependent manner with an IC_50_ of 0.34 mg/ml as the keys indicated, which was used to simulate the effect of XSN on human AP. **(C)** Shows the comparative effect of XSN and liensinine on APD (Ma et al., 2020).

Studies using patch clamp electrophysiological methods to study the effects of XSN on action potential (AP) and ionic channels of isolated cardiac myocytes have discovered, as illustrated in Figure 1, that XSN is not only a potassium channel regulator but also a sodium channel blocker (Ma et al., 2017; Wang et al., 2018). It could prolong the duration and suppress the amplitude of AP, indicating the inhibition of sodium and potassium channels, hence inhibiting the reentrant arrhythmia by increasing the effective refractory period. The effect of XSN is similar to that of amiodarone and other class I and III antiarrhythmic drugs (Ma et al., 2020).

The blockade effects of XSN on sodium and potassium channels of the isolated rat cardiac ventricular myocytes were further observed in expressed human hNav1.5 and hERG channels (Wang et al., 2019c).

### 2.4 The Effect of XSN on the Myocardium Conduction Properties of Human iPSC-Derived 2D Myocardial Tissue Studied in a Multi-Electrode Array System

It was recently reported that XSN significantly reduced the velocity of the myocardial conduction at concentrations higher than 0.4 g/L and significantly suppressed the amplitude of the initial phase of field action potential (FAP) conducted by Na^+^. At 1.6 g/L, XSN prolonged the duration of Na^+^ conduction, and at concentrations higher than 0.1 g/L, inhibited the maximum velocity of Na^+^ conduction. The myocardial beating interval was significantly prolonged, and the rate of the beating also decreased under all the concentrations of XSN applied. Furthermore, XSN also prolonged FAP duration even at the lowest concentration applied (0.025 g/L) and decreased the FAP amplitude significantly at the highest concentration studied (Wang et al., 2021).

### 2.5 Xin Su Ning Protects the Heart From I/R-Induced Injury

**FIGURE 2:**
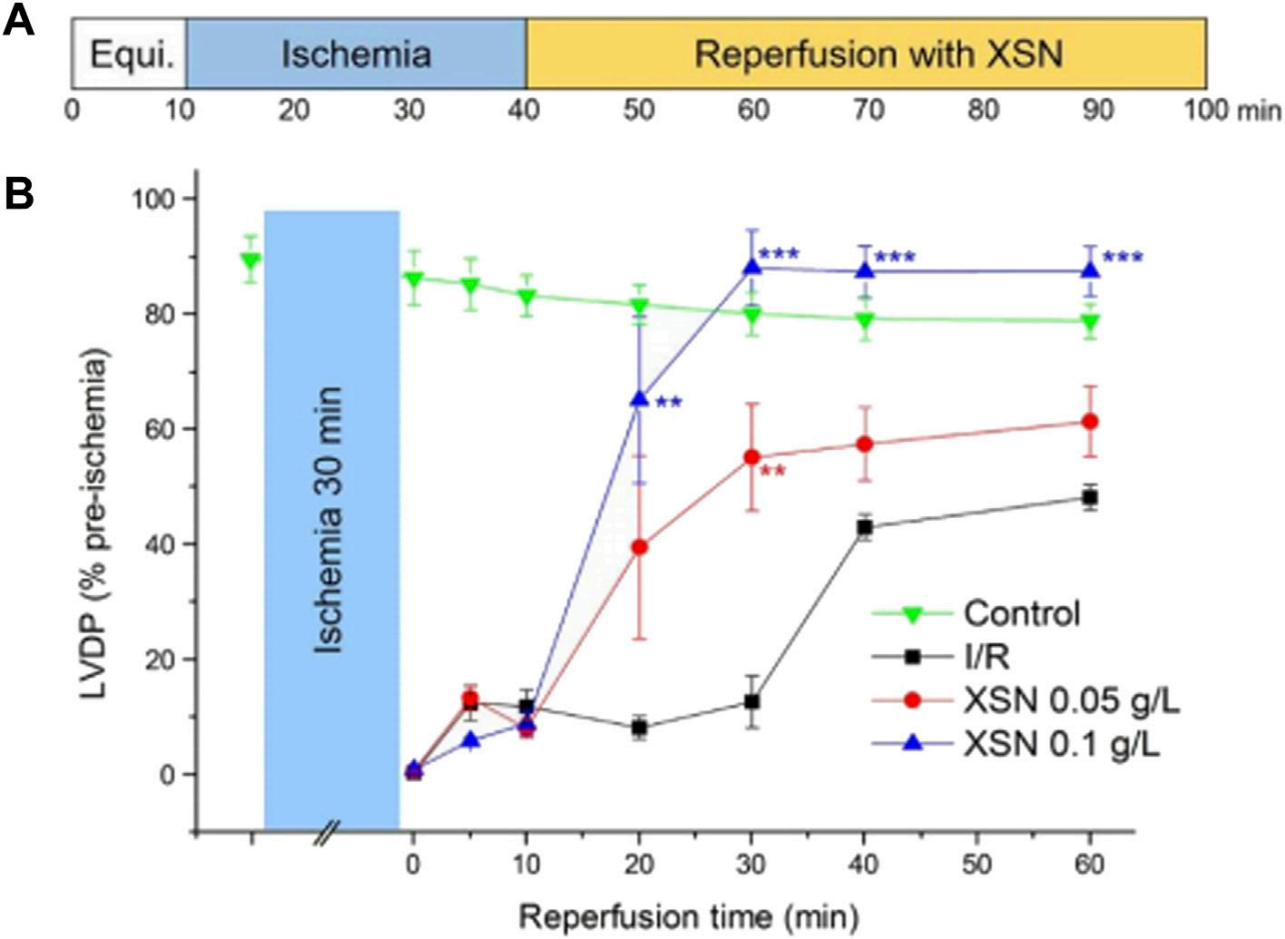
Protective effect of XSN on ischemia/reperfusion-induced injury of the heart. **(A)** Protocol of contractile function of an I/R perfused rat heart; **(B)** % rate of LVDP of rat hearts after XSN treatment versus control (Wang et al., 2018).

The cardio-protective effect of XSN in I/R-induced injury in the isolated heart was evaluated. As demonstrated in Figure 2, XSN produced significant recovery of left ventricular diastolic pressure (LVDP) in comparation with the control. XSN’s role in improving cardiac systolic function on the I/R injured rat heart was achieved by increasing LVDP, rate-pressure product (RPP), max dP/dt, and min dP/dt. These protective effects may contribute to the antiarrhythmic effect of XSN (Wang et al., 2018).

### 2.6 The Effect of the Active Components of Xin Su Ning on hERG and hNav1.5 Channels

Several isolated components of XSN were studied to identify the likely contributions that they may make to the antiarrhythmic action of XSN. Among the components tested, liensinine, from Lianzixin (*Plumula nelumbinis*), one of the 11 herbs in XSN, was speculated to be an important component that affects the electrophysiological properties of cardiac myocytes. However, liensinine showed APD prolongation action with a different repolarization profile than XSN; liensinine had effects on APD_90_ only, whereas XSN impacted both APD_90_ and APD_50_ significantly (Figure 1B), which illustrated well the complexity of XSN as a multicomponent medicine in exerting its clinical efficacy (Ma et al., 2020).

The effect of liquiritigenin and isoliquirigenin, compounds in GanCao (one of the 11 herbs formulating XSN), were reported to be active antiarrhythmic components: isoliquirigenin blocks both hERG and Nav1.5 channels, while liquiritigenin blocks Nav1.5 channels, which indicates that both compounds would contribute to the class I and III antiarrhythmic action of XSN (Wang et al., 2019a; Sweeney et al., 2019; Mujahid et al., 2020)**.**

## 3 Clinical Studies of Xin Su Ning

### 3.1 Pre-Registration Clinical Studies

Based on clinical study authorization number (1999) ZL-12 issued by the China State Drug Administration, XSN capsule was used to treat 300 cases of premature ventricular contraction with PHHD syndrome from August 2000 to January 2003 by the First Affiliated Hospital of Anhui University of Traditional Chinese Medicine, Shandong Provincial Hospital, Qilu Hospital of Shandong University, and Shaanxi Provincial Hospital of Traditional Chinese Medicine. The comparator medicine used in the study was Xin Lv Ning tablets.

The total effective rate on premature ventricular contraction (PVC) with the XSN group was 53.7% compared with the control group’s rate of 42.9% (*p* = 0.044). The total effective rate in symptomatic relief with the XSN group was 66.0% compared with the control group’s rate of 54.3% (*p* = 0.024). The XSN group also significantly relieved the symptoms of palpitation (*p* < 0.05). The onset time of treatment of chest tightness, insomnia, and dreaminess in the XSN group was shorter than that in the control group (*p* < 0.05). The results of safety studies showed that the XSN capsule had no adverse effects on blood, urine, stool routine, and heart, liver, and kidney functions, which indicated that XSN is an effective and safe medicine for treating PVC arrhythmia in humans.

### 3.2 Other Clinical Case Studies

In 2011, a study reported a total effective rate of 88.5% in 26 cases of frequent PVCs treated with XSN (Lin et al., 2011). In another study in 2008, the efficacy evaluation of XSN in the treatment of 30 viral myocarditis cases was reported; the results showed that the total effective rate was 83%, which was significantly more than that of the control group (*p <* 0.05) (Wang and Lu, 2008). In addition, the study published in 2000 by the Affiliated Hospital of Shandong University of Traditional Chinese Medicine evaluated XSN in a 90-patient trial (Yuan and Zhou, 2000). The clinical curative effects of XSN were observed and compared with those of propafenone and atenoloum (PRO.&ATL.). The study demonstrated an overall efficacy of 85% with XSN in treating arrhythmia, similar to that demonstrated in the PRO.&ATL groups. Besides, the XSN group had a better effect on improving the overall conditions in patients with PVCs complicated with coronary heart disease, but PRO.&ATL groups did not show the same therapeutic benefit. XSN displayed clinical effects on atrial and ventricular premature beats, atrial fibrillation, paroxysmal supraventricular tachycardia, and sinus tachycardia, and the curative effect on mild and moderate arrhythmias was better than that on severe arrhythmias. At the same time, it can also reduce the blood lipid level of some patients with hyperlipidemia, improve their hemorheological indexes, and change the state of coronary insufficiency and cardiac function in some patients (Yuan and Zhou, 2000).

### 3.3 Randomized Controlled Trial

The results of a three-armed, randomized, double-blind, placebo-controlled, parallel-group, multicenter trial of XSN were reported recently (Ma et al., 2020). This trial was registered in the Chinese Clinical Trial Register Center (ChiCTR-TRC-14004180) and ran between April 2014 and January 2016 across 39 hospitals in mainland China. The study enrolled 861 eligible patients randomly assigned in a ratio of 2:2:1 to receive XSN (XSN four capsules, 0.48 g per capsule plus simulated mexiletine two pills, n = 343), mexiletine (mexiletine two pills, 50 mg per pill plus simulated XSN four capsules, n = 345), or the placebo (simulated XSN four capsules plus simulated mexiletine two pills, n = 173). Each participant took the treatments three times per day for 4 weeks. At the end, a total of 779 (90.48%) patients (307 in the XSN group, 320 in the mexiletine group, and 173 in the placebo group) completed the 4-week follow-up and were included in the final efficacy analyses (Zhai et al., 2017).

**FIGURE 3:**
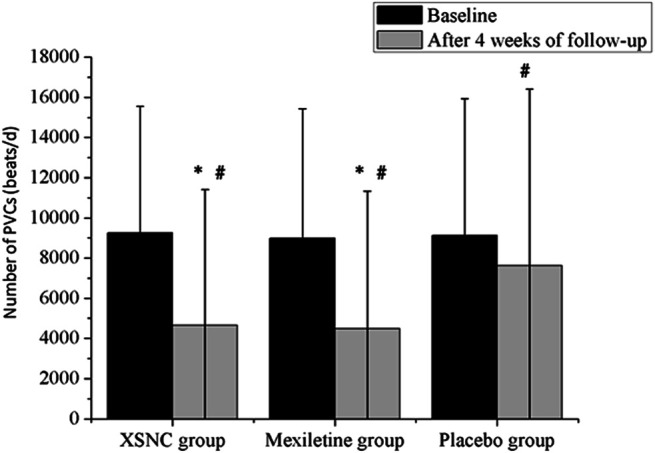
Total number of PVCs from baseline to 4 weeks after treatment in the trial groups. PVCs, premature ventricular contractions. #*p* < 0.001 vs. baseline, *p < 0.001 vs. the placebo group. Continuous data were presented as the mean ± standard deviation (SD). A smaller number of PVCs were observed after a 4-week treatment than at baseline, in the XSN group (4645.89 ± 6,772.17 vs. 9,250.56 ± 6,297.37 beats/d, *p* < 0.0001), the mexiletine group (4480.37 ± 6,851.37 vs. 8,983.23 ± 6,439.02 beats/d, *p* < 0.0001), and the placebo group (7,617.16 ± 8,794.66 vs. 9,129.63 ± 6,796.15 beats/d, *p* < 0.0001). In addition, compared to the placebo group, the XSN group and the mexiletine group had a statistically significant change in the total PVC frequency after the 4-week treatment period (*p* < 0.0001) (Ma et al., 2020).

The PVC numbers at baseline were not significantly different between the XSN group, the mexiletine group, and the placebo group (*p* = 0.5886). In comparison to the placebo group, the XSN group and the mexiletine group both had a statistically significant change in the total PVC frequency (*p* < 0.0001, Figure 3).

In the study, it was found that XSN improved the overall counts of PVCs and PVC-related symptoms in a pre- and posttreatment analysis, and its efficacy was noninferior to that of mexiletine, a class Ib conventional anti-arrhythmic drug (AAD), and was significantly superior to that of the placebo. In the safety set analysis, adverse events were reported in 50 patients, including 18 in the XSN group, 21 in the mexiletine group, and 11 in the placebo group (*p* = 0.8430). Neither death nor serious adverse events related to XSN were reported during the study. Compared with the placebo group, the administration of XSN had no significant impact on the liver, kidney functions, and the ECG parameters (Table 1). The result from this randomized controlled trial aligns with more than 30 years of clinical use of XSN in China (Ma et al., 2020).

**TABLE 1:** Laboratory evaluations and ECG parameter changes between the two groups, medium (range) (Ma et al., 2020).

Variables	XSN group		Mexiletine group		Placebo group	
	0 weeks	4 weeks	0 weeks	4 weeks	0 weeks	4 weeks
Laboratory tests						
ALT, IU/L	33 (4–41)	21 (5–40)	38 (9–50)	32 (7–48)	21 (8–42)	22 (8–43)
AST, IU/L	27 (4–40)	18 (21–72)	21 (5–40)	18 (17–59)	20 (6–45)	23 (7–53)
Scr, mmol/L	71 (62–106)	69 (60–106)	60 (53–97)	61 (44–105)	51 (44–104)	56 (44–106)
ECG parameters						
QT interval, ms	402 (280–464)	399.5 (297–442)	405 (297–493)	406 (318–483)	397 (326–492)	402 (337–487)
Mean HR, bpm	73 (67–103)	73 (67–96)	74 (67–89)	73 (68–89)	72 (67–99)	72 (64–93)
Maximum HR, bpm	114 (102–127)	115 (101–127)	114 (103–128)	114 (102–126)	112 (100–124)	114 (102–126)
Minimum HR, bpm	51 (47–75)	51 (46–86)	51 (47–75)	52 (47–71)	52 (36–78)	51 (36–75)

ALT, alanine aminotransferase; AST, aspartate aminotransferase; Scr, serum creatinine; ECG, electrocardiogram; HR, heart rate; XSN, Xin Su Ning.

## 4 The Measurable Chemical Compounds in Xin Su Ning

The main chemical compositions contained in the XSN capsule were studied by silica gel column chromatography, octadecyl silane (ODS) column chromatography, Sephadex LH-20 column chromatography, reversed-phase high performance liquid chromatography (RP-HPLC), and other chromatographic separation methods (Zhang, 2017a). Overall, 22 monomer compositions were separated, and their structures were identified. These compounds are berberine, daucosterol, sophora flavescens chalcone, coptisine, dihydroberberine, dihydrocoptisine, dihydrocoptisine, hesperidin, jateorhizine, isoliquiritigenin, Kushenol O, sophocarpidine, sophocarpine, coumarin, N-trans-feruloyltyramine, liquiritigenin, nobiletin, hesperetin, 8-oxyberberrubine, 4-(2-hydroxy-vinyl)-benzene-1,2-diol, 5-demethenyl nobiletin, scopoletin, and β-sito-sterol. Among them, five chemical compositions are from Huanglian, four from Zhishi, four from Kushen, two from Gancao, and the remaining seven from other medicinal materials.

**FIGURE 4:**
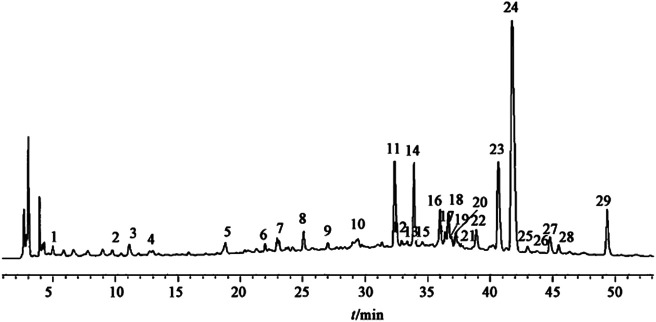
HPLC fingerprint and identification of peaks of XSN capsules. (Zhang et al., 2017).

As shown in Figure 4, there were 29 common peaks in different batches of the XSN capsule, and 12 peaks were identified (Zhang et al., 2017). The identified peaks were peak No. 2 (sophoridine), peak No. 14 (scopoletin), peak No. 11 (scopoletin), peak No. 1 (hesperidin), peak No. 14 (neohesperidin), peak No. 16 (epiberberine), peak No. 17 (Dihydroberberine), peak No. 18 (jatrorrhizine), peak No. 19 (berberine), peak No. 21 (Dihydroberberine), peak No. 23 (palmatine), peak No. 24 (berberine), and peak No. 29 (glycyrrhizic acid). Among them, peaks 5, 8, 16, 17, 19, 21, 22, 23, 24, 25, and 27 belong to Huanglian, peaks 10, 11, and 14 belong to Zhishi, peaks 20, 28, and 29 belong to Gancao, peak 1 belongs to Banxia, peak 3 belongs to ginseng, peak 9 belongs to Qinghao, peak 26 belongs to Changshan, peak 2 belongs to Kushen, peak 4 may come from Banxia, Qinghao, Ginseng, and Gancao, peak 5 may come from Huanglian, Changshan, and Qinghao, peak 6 can be from Changshan or Lianzixin, peak 7 can be from Huanglian or Zhishi, and peak 12 can be from Kushen or Maidong. The analytical method of HPLC fingerprint is stable and reliable with fine repeatability, which provides a reference for study on a material basis and quality control of the XSN capsule.

In another study, ultrahigh-pressure liquid chromatography coupled with linear ion trap-Orbitrap tandem mass spectrometry (UHPLC-LTQ-Orbitrap) was used on XSN. 41 compounds were identified that may contribute to the therapeutic effects of XSN. These data showed that berberine, palmatine, scopoletin, liquiritigenin, naringenin, formononetin, nobiletin, tangeretin, 5-demethylnobiletin, kushenol E, and kurarinone might function as candidate markers for qualitative evaluation of XSN (Guo et al., 2018).

## 5 Pharmacology Network Study of Xin Su Ning

TCM is one of the well-known traditional medicines used and developed through a thousand years of human history with the distinguished feature of a multi-herbal/multicomponent nature, which has been a major obstacle to evaluating its clinical efficacy using conventional pharmacological methods. Along with the development of computational simulation of biological activities, computational network pharmacology opens up possibilities for predicting the pharmacological actions of multicomponent TCM. However, the network pharmacology approach has been taking all the components in a formula in equal weighting, ignoring the relative proportion of each component in the analysis. Since the relative proportion of each herb is critical in terms of clinical outcome guided by the herbal formulation theory of Chinese medicine, a novel approach which takes into account the relative proportion of different herbs has been introduced to evaluate the potential pathways of the antiarrhythmic TCM XSN. This was done by introducing a novel parameter, a weight coefficient, to calculate the concentrations of all the measurable compounds in XSN. This allowed for the creation of a more realistic concentration-related pharmacological panorama linked with the syndromes of PHHD arrhythmia (Wang et al., 2019b).

In this study, a total of 963 monomer components from the 11 herbs of XSN were collected from Chemical Components of Source Plants in Traditional Chinese Medicine and the TCMSP database. Statistics relating to the chemical properties found in the 11 herbs and XSN’s target spectrum were summarized. The contents of 47 of the 963 components were quantified, with the rest set to the lowest value in analysis.

**FIGURE 5:**
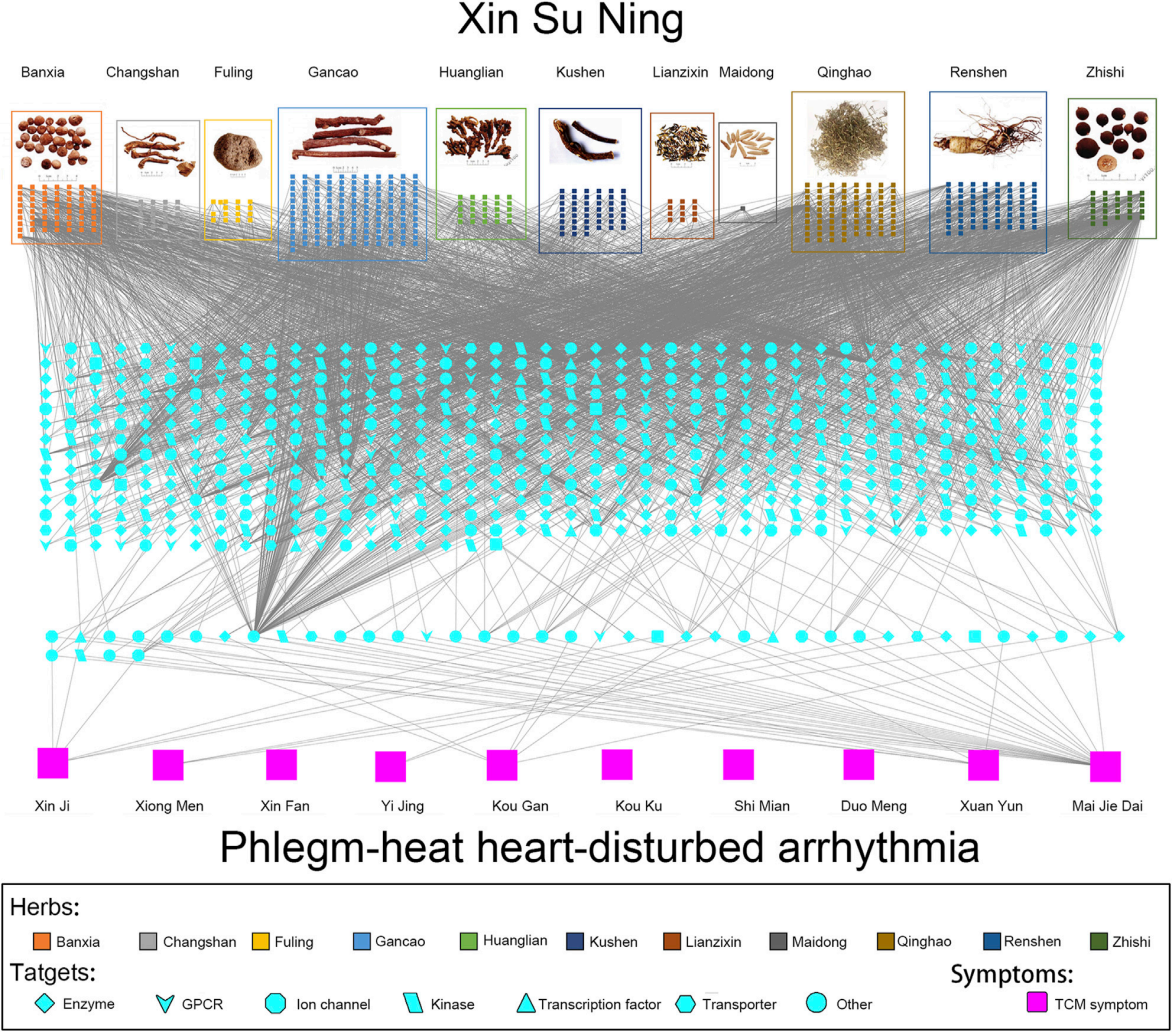
Panoramagram of the pharmacological mechanism of XSN for treating PHHD arrhythmia. **(A)** 11 herbs that formulated XSN are shown in the box with different colors, and the components of each herb are shown in small squares with the same color as their source herb. **(B)** Target spectrum of XSN. All the targets were corresponding to the component in panel A; the 41 targets showed in the dash line box in the bottom are the overlapping target set between the XSN target spectrum and the PHHD-arrhythmia target spectrum. Targets were shown in different shapes according to the classifications as indicated in the keys. **(C)** Arrhythmia with Zheng and relative TCM symptoms. Ten TCM symptoms of PHHD arrhythmia were represented by large pink squares in the bottom panel (Wang et al., 2019b).

The pharmaconetwork analysis also reviewed the relationship between Zheng (TCM syndrome), which is represented by the series of characteristics with clinical manifestations and symptoms and all target genes. There are 10 TCM symptoms of arrhythmia relating to PHHD: Xin Ji (palpitations), Xiong Men (respiratory distress), Xin Fan (boredom), Yi Jing (panic attack), Kou Gan (xerostomia), Kou Ku (bitter taste in the mouth, not bad breath in the mouth), Shi Mian (insomnia), Duo Meng (dreaminess), Xuan Yun (vertigo), and Mai Jie Dai (includes two types of pulses; Jie Mai is knotted or bound pulse, which is slow and relaxed and stops at irregular intervals. Dai Mai means the pulse displays regularly intermittent abnormality). Hence, the panoramic view of the integrative pharmacological mechanism of XSN is interpreted in Figure 5. This panoramagram elaborates the relationships between the XSN formula, herbs, components, targets, symptoms, and PHHD arrhythmia; the pharmacological network consisted of 963 components, 618 targets, and 10 symptoms.

The evidence obtained so far demonstrated that XSN exerts an impact on PHHD syndrome arrhythmia through both fast-acting and long-acting mechanisms. On the fast-acting mechanism, XSN regulates multiple ion channels. On the long-acting mechanism, XSN protects the heart from ischemia reperfusion injury, inhibits the apoptosis of cardiomyocytes, and improves glucose and lipid metabolism.

## 6 Theories of TCM and Principles of Xin Su Ning Formulation

### 6.1 Discussion of TCM Theories on Tachyarrhythmia

Even though the term arrhythmia was not coined in ancient TCM books, symptoms of arrhythmia were widely recorded. The earliest descriptions of arrhythmia symptoms could be traced to “Su Wen: Zhi Zheng Yao Da Lun” and “Ling Shu: Ben Shen” and included the words “Palpitation with emptiness,” “Fear and anxious,” and “The heart meridian does not flow smoothly, causing a feeling of anxiousness and abnormal heartbeat.” “Su Wen: Ping Ren Qi Xiang Lun” said “Pulse beats irregularly will cause death,” which was the earliest explanation of arrhythmia-induced sudden death. Although the exact date is unknown, it is widely believed that the book of Su Wen and Ling Shu was written more than 2000 years ago in the Western Han Dynasty (Hebei Medical College, 2009; Wang, 2016).

Simple analogies using Western medicine is not adequate in explaining the full meaning behind the heart, phlegm, and heat. The heart relates to mental behavior and the neurological state of mind. Therefore, when the heart “spirit” is disturbed, patients have symptoms such as insomnia, anxiety, and palpitation. The concept Heart in Chinese medicine mainly refers to the circulatory system and the nervous system, and they are easily blocked or obstructed to cause malfunction (Jiao et al., 2015). This is why PHHD is classified as an “excess” syndrome; the heat and the phlegm must be cleared to treat the symptoms in the circulatory system and the neuronal system successfully.

Heat is a state of the body, where if an individual is in the heated state, the body manifests inflamed ulcers, a red-colored tongue, swollen body parts, dry stools, sore throats, and a series of other symptoms particularly associated with inflammation.

Jin-ye fluids are bodily liquids other than blood. This may include urine, sweat, saliva, and gastric juices. Disturbance to Jin-ye would lead to a range of physiological responses that produce phlegm, similar to mucus generation. Phlegm can be considered an imbalance to homeostasis. Based on TCM theories, if phlegm-heat is stuck at the throat, then the patient would suffer from a sore throat. If the phlegm is stuck at the chest, then the patient would suffer from chest pain, chest tightness, and nausea. If phlegm-heat obstructs the mind, it would also obstruct the heart Qi, causing irregular pulse rhythm and incongruity. Therefore, phlegm-heat culminates in disturbing the heart, and the resulting symptoms are palpitation, panic, restlessness at night, mental disorder, a yellow greasy tongue, and other manifestations. Hence, phlegm-heat is responsible for various kinds of diseases, affecting many internal organs. There are many records about the pathogenesis of arrhythmia in ancient literature. For example, “Qian Jin Yi Fang” says: “Irregular heart beat is caused by phlegm-heat” (Sun, 2014).

Therefore, treatments for different symptoms need to be matched to their respective original disturbances. Every Chinese herb has its own unique traits, which have different impacts on these disturbances. For example, Huanglian is cold in nature, and one of the actions of Huanglian is to regulate heart function. Clinically, patients’ symptoms are likely to be complex in nature, with roots of the illnesses implicating multiple organs. As a result, the herbs in a formula are composed in an orderly manner according to the Emperor–Minister–Adjuvant–Courier configuration. Each herb exerts its impact through its unique attributes, and the synergistic effects produce higher efficacy (than a single herb or compound does) and lower side effects.

### 6.2 History and Components of Xin Su Ning

In the 1980s, most of the treatments of arrhythmia with TCM were based on treating a lack of Qi, Qi deficiency. General signs of deficiency are fatigue, frail and weak movement, paleness, and incomplete engagement with life. Qi is a broad term in Chinese culture. In the context of clinical medicine, it means the dynamics of producing energy. However, nowadays, there are great changes in our living environment including natural and social environment, living conditions, dietary habits, etc. (Ding et al., 2018). The high-fat and high-sugar diet, coupled with reduced physical activity, would affect the normal operation of Jin-ye and cause the accumulation of pathological phlegm and dampness in the body. The high-intensity nature of the modern work environment makes the spirit prone to nervousness, which would affect the mind and make it more likely to generate the PHHD syndrome. Arrhythmia with typical clinical manifestation of this kind of syndrome takes a large proportion of the clinical treatment, and as far as we know, XSN is the only medicine for treating PHHD arrhythmia (Yuan and Zhou, 2000).

XSN is adapted from Huanglian Wen Dan Decoction recorded in Tingzhen Lu’s “Liu Yin Tiao Bian” in the Qing Dynasty (Lu, 1982), which has the effect of removing phlegm and heat. Huanglian, Banxia, Fuling, Gancao, and Zhishi are from Huanglian Wen Dan Decoction; Changshan, Lianzixin, Qinghao, and Kushen were added to strengthen the antiarrhythmic effect of XSN. PHHD is classified as heat persisting in the body for a long time without dissipating, which leads to fluids concentrating as phlegm (Jiao et al., 2015). Common PHHD symptoms are palpitation, distress, insomnia and dreamful sleep, dry stool, red tongue (the tongue body is red, especially at the tip, which is often swollen and painful) with a yellow and greasy coating (Bi, 2015; Zhang et al., 2020), and knotted or regularly intermittent pulse. Depending on the severity of phlegm heat interfering with the nervous system, some mental symptoms may also occur, such as boredom or even irritability (Ren et al., 2012; Xing et al., 2015). Prevalence of PHHD has risen in recent years due to changing lifestyles, and XSN is designed specifically to treat this type of arrhythmia.

The prescription aims to clear away heat and phlegm and soothe the heart and palpitations. Therefore, XSN, developed from Huanglian Wen Dan Decoction, has demonstrated significant clinical benefit, particularly relating to the PHHD type of arrhythmia.

The antiarrhythmic pharmacological properties obtained through modern research of each herb is summarized in Table 2. Each herb of XSN will be further discussed below. However, XSN is not simply composed of herbs with antiarrhythmic properties. A good Chinese medicine formula conducts “Emperor–Minister–Adjuvant–Courier” principles so as to produce the best clinical efficacy and minimal side effects. Therefore, every herb in XSN is essential in producing the clinical efficacy.

**TABLE 2:** Antiarrhythmic properties of the herbal medicines that formulated XSN.

Herbs	Direct and indirect antiarrhythmic properties	References
Coptidis Rhizoma (Huanglian, *Coptis chinensis* Franch.)	Prolonging APD and the effective refractory period, eliminating the reentry impulse, and displaying significant antiarrhythmic effects. Promoting extracellular calcium influx, increasing intracellular calcium, improving sinus node function, and changing cardiac conduction from the unidirectional block to the bidirectional block. Inhibiting the activity of cholinesterase, enhancing the effect of acetylcholine, and increasing the membrane potassium conductance and the intracellular potassium outflow	Wang and Lu (2008); Xu and Gu (2017); Siqin et al. (2014); Dai and Luo (2001); Qiu (2018); Yang (2008)
Pinelliae Rhizoma (Banxia, *Pinellia ternata* [Thunb.] Makino)	Quinidine-like anti-arrhythmia effect. Banxia extract has significant inhibition effect on PVC in dogs caused by barium chloride. Increasing coronary flow and amplitude of the cardiac contraction curve of the isolated rabbit heart	Ding et al. (2018); Teng et al. (1983); Teng et al. (1985); Liu and Li (1997)
Poria (Fuling, *Poria cocos* [Schw.] Wolf)	Improving urinary retention and cardiac function in chronic heart failure rat models, inhibiting the occurrence of cardiac hypertrophy, improving hemodynamics, enhancing myocardial contractility, and improving myocardial diastolic function and cardiac output	Li et al. (2015a); Yang (2012)
Aurantii Fructus Immaturus (Zhishi, *Citrus aurantium* L.)	Zhishi can enhance myocardial contractility and increase cardiac output and coronary flow	Yang (2007); Zhang et al. (2009); Han et al. (2010); Xu et al. (2009); Huang et al. (2012)
Dichroae Radix (Changshan, *Dichroa febrifuga* Lour.)	Intravenous injection of Changshan alkali into anesthetized dogs can reduce the amplitude of cardiac contraction. Changrolin has quinidine-like anti-arrhythmia effect. The combination of Qinghao and Changshan has protective effect on arrhythmia and shortens the duration and frequency of arrhythmia	Zhang and Huang (1956); Yu et al. (1997); Chen et al. (2010); Fan et al. (2020); Jiao (2006); Liu (2007)
Nelumbinis Plumula (Lianzixin, *Nelumbo nucifera* Gaertn.)	The alkaloids in Lianzixin can shorten the recovery time of the sinus rhythm and reduce the incidence of ventricular fibrillation in mice induced by chloroform and delay the occurrence time of ventricular fibrillation in mice. Liensinine and neferine can inhibit the transmembrane transport of Na+, Ca2+, and K+ and produce synergistic antiarrhythmic effect. Isoliensinine may antagonize stretch-induced arrhythmia by blocking stretch-activated ion channels	Zeng et al. (2007); Wang et al. (1992); Dong et al. (2012); Chen et al. (2006)
Sophorae flavescentis Radix (Kushen, *Sophora flavescens* Ait.)	Kushen alkaloid compounds have anti-atherosclerosis effect. It has been reported to have dilating effects on the coronary artery and peripheral blood vessels, increase myocardial oxygen supply, and reduce myocardial oxygen consumption. Matrine has an effect on arrhythmia induced by low calcium and low magnesium. Matrine is effective on arrhythmia after myocardial infarction. Oxymatrine can effectively prolong the QT interval and the effective refractory period, increase the excitability threshold of the myocardial diastolic phase, promote the decrease in myocardial cell autonomy and triggered activity	Chen et al. (2018); Chen et al. (2016); Li et al. (2012); Sui et al. (2008); Zhang et al. (2008); Li et al. (2015b); Wang and Liu (2011); Chen et al. (2018)
Artemisiae annuae Herba (Qinghao, *Artemisia annua* L.)	Qinghao can significantly inhibit arrhythmia in rats induced by coronary ligation, electrical stimulation, or aconitine. It can also protect the heart from myocardial ischemia induced by pituitrin in rats and accelerate the heart rate. Artemisinin could inhibit the release of intracellular Ca2+ by interfering with the outward rectifying K+ current in ventricular myocytes, and it can significantly prolong the atrial effective refractory period and shorten the duration of atrial fibrillation in rats	Shi (2015); Qiao et al. (2007); Wang and Pei (2020); Li et al. (2020); Reid et al. (2016)
Ginseng Radix et Rhizoma (Renshen, *Panax ginseng* C. A. Mey.)	Ginsenosides can inhibit arrhythmia by affecting potassium and calcium channels and protecting myocardial cells from injury caused by ischemia reperfusion or chemical reagents. Ginsenoside Rb1 significantly inhibited the L-type calcium current and the transient outward potassium current in ventricular myocytes. Ginsenoside Re can inhibit the voltage-dependent sodium channel current and the transient outward potassium channel current in ventricular myocytes. Ginsenoside Rg3 can slow down the apoptosis of rat cardiomyocytes and protect the myocardium	Chen et al. (2015); Pei et al. (2011); Yong et al. (2013); Zhang et al. (2013); Wang et al. (2015b); You and Tian (2021)
Ophiopogonis Radix (Maidong*, Ophiopogon japonicus* (L.f) Ker Gawl.)	Maidong may have antiarrhythmic effects by improving myocardial blood supply. Maidong saponins can reduce the excitability and automaticity of the right atrium muscles and prolong the refractory period of the left atrium. Maidong polysaccharide can enhance the tolerance of myocardial ischemia and hypoxia, increase coronary flow, protect ischemic myocardial cells, promote the proliferation and differentiation of endothelial progenitor cells in rats with myocardial ischemia-reperfusion, repair the ischemic myocardium, and restore cardiac function	Yu et al. (2014); Yu et al. (2014); Xin et al. (2011)
Nardostachyos Radix et Rhizoma (Gancao, *Glycyrrhiza uralensis* Fisch.)	Liquorice can inhibit arrhythmias caused by aconitine and ouabain. It can also shorten the time of arrhythmias induced by BaCl2 in rats and significantly slow down heart rates. It can protect the myocardium and has obvious anti–myocardial ischemia activities. Gancao can reduce ectopic pacing and improve ECG conduction	Pan (2004); Zhang et al. (2015); Xu and Zhang (1997)

It should be noted that a large number of ancient books of TCM are cited as references in this study. The year of references is the year when modern scholars reorganized the manuscripts and published them, which does not represent the years that ancient books were written in. Take this sentence as an example: “Ben Cao Gang Mu” recorded that “Huanglian could cure palpitation (Tang, 2009).” “Ben Cao Gang Mu” was written by Shizhen Li of the Ming Dynasty in 1578 AD. The book contains 52 chapters, including 1892 herbal medicines (including a small number of other types of medicine) and more than 10,000 prescriptions. It is a collection of achievements of pharmacy before the 16th century in China.

#### 6.2.1 Coptidis Rhizoma (Huanglian, *Coptis chinensis* Franch.)

Huanglian is the rhizome of *Coptis chinensis* Franch., *C. deltoidea* C. Y. Cheng et Hsiao, or *C. teeta* Wall (Ranunculaceae) (Liu et al., 2017). Huanglian is bitter in taste and cold in nature. One of the actions of Huanglian is to regulate heart function. It could clear heat and dry dampness. “Ben Cao Gang Mu” recorded that “Huanglian could cure palpitation (Tang, 2009).” Therefore, the ancients used Huanglian to treat jaundice, high fever, and dental ulcers and soothe palpitation, chest pain, and anxiousness and insomnia, such as Huanglian An Shen Decoction in “Zhizhi Fang” (Wang, 2014).

Huanglian mainly contains berberine, jatrorrhizine, palmatine, epiberberine, and coptisine. The content of total alkaloids is as high as 70–80%. In addition, there are lignans, phenolic acids, flavonoids, volatile oils, and polysaccharides and some trace elements necessary for the human body (Wei et al., 2014; Wang L. et al., 2015).

Modern pharmacological studies have shown that Huanglian not only has broad-spectrum antimicrobial and antiviral effects but also has the effects of reducing myocardial autonomy, prolonging APD and the effective refractory period, eliminating reentry impulse and displaying significant antiarrhythmic effects, improving outcomes of heart failure and anti-myocardial ischemia, and improving myocardial microcirculation in the treatment of cardiovascular and cerebrovascular diseases (Wang and Lu, 2008; Xu and Gu, 2017). Berberine is the main component of Huanglian (Wang et al., 2014). Previous studies and clinical reports have shown that berberine has significant antiarrhythmic effects on arrhythmias with different etiologies (Siqin et al., 2014). Importantly, berberine was demonstrated to increase intracellular calcium through extracellular calcium influx, improving sinus node function (Dai and Luo, 2001). Berberine can selectively antagonize ouabain-induced ventricular arrhythmia in animals, and its antiarrhythmic effect may be related to the inhibition of Na^+^ influx in the myocardium (Qiu, 2018). Berberine has been demonstrated to prevent or treat experimental ventricular arrhythmias caused by CaCl_2_, adrenaline, ouabain, aconitine, electrical stimulation, and coronary artery ligation. Its antiarrhythmic mechanism may be related to the reduction of myocardial automaticity, prolongation of APD and the effective refractory period, and elimination of the reentry impulse. Berberine can significantly inhibit atrial, ventricular premature beat, supraventricular tachycardia, and other arrhythmias (Yang, 2008). Besides, palmatine is another major component of Huanglian. The content of total alkaloids is second only to that of berberine, accounting for 1.28–2.12% (Zhou and Shen, 2004), and palmatine hydrochloride can inhibit arrhythmia induced by chloroform, adrenaline, and ouabain in rats. In addition, palmatine was also reported to have an antagonistic effect on chloroform-induced atrial fibrillation in mice (Chen and Fang, 1992).

#### 6.2.2 Pinelliae Rhizoma (Banxia, *Pinellia ternata* [Thunb.] Makino)

Banxia is the root of *Pinellia ternate* (Thumb). Breit., which belongs to the Pinellia genus of Araceae (Wang and Wang, 2020). Banxia is spicy in taste, warm in nature, and promotes the health of the digestive system and lungs. Its functions include eliminating dampness and dissipating phlegm, eliminating swelling and dispersing stagnation, and treating phlegm-rich cough, chest stuffiness, dizziness, palpitation, etc. “Ben Cao Gang Mu” recorded that “In addition to treating abdominal distension, it is also used for dizzy and eye blurry (Tang, 2009).” “Ben Jing:” “The main use of Banxia is to treat chills and fever, sore throat, dizziness, chest swelling and cough (Mo, 2015).” The main active components of Banxia are alkaloids: ephedrine, choline, etc. In addition, Banxia aqueous solution contains a variety of nucleosides: guanosine, thymidine, adenosine, cytidine, uridine, etc. Banxia also contains aromatic components such as vanillic acid, caffeic acid, ferulic acid, *p*-hydroxycinnamic acid, and urine black acid. In addition, there are volatile oils, long chain fatty amino acids, and polysaccharides (Kurata et al., 1998).

Pharmacological studies have revealed that Banxia has anti-inflammatory and anticancer properties. The Ningxin alkaloid component of Banxia has quinidine-like antiarrhythmic effect (Ding et al., 2018). Some studies have shown that intravenous injection of Banxia extract (equivalent of 0.2–0.3 g crude drug/kg) has significant effect on PVCs in dogs caused by barium chloride; in 39 out of 40 studies, after administration of Banxia extract, the PVCs were rapidly inhibited without recurrence, showing an effective rate of 98% (Teng et al., 1983; Teng et al., 1985). In another study, the average duration of PVCs induced by barium chloride was shortened from 81 to 39 min by intragastric administration of Banxia water extract in rats. In addition, studies have shown that Banxia can increase coronary flow and amplitude of the cardiac contraction curve of an isolated rabbit heart (Liu and Li, 1997).

#### 6.2.3 Poria (Fuling, *Poria cocos* [Schw.] Wolf)

Fuling is the dried sclerotia of the fungus *Poria cocos* (Schw.) Wolf. It was first recorded in “Shen Nong Ben Cao Jing” in the Han Dynasty. Its taste is light and sweet, and it is mild in nature. It promotes the health of the heart, lungs, digestive function, and kidneys. It is mainly used for the treatment of phlegm, palpitation, diarrhea, restlessness, and insomnia (Wu, 2016).

Fuling is made of predominantly beta-pachyman, which accounts for about 93% of the dried products. It also contains fatty acids, such as lauric acid, palmitic acid, and dodecanoic acid, as well as ergosterol, lecithin, and other inorganic components. Fuling is widely used in dysuria and edema (Wu et al., 2014). Studies showed that Fuling could downregulate the expression of aquaporin 2 (AQP2) mRNA and protein, reduce the excretion of AQP2 in urine, and downregulate the expression of arginine vasopressin in plasma and expression of mRNA of vasopressin receptor 2, thereby improving urinary retention and cardiac function in rats with chronic heart failure. Fuling polysaccharide can inhibit the occurrence of myocardial hypertrophy, improve hemodynamics, enhance myocardial systolic function, improve myocardial diastolic function, and increase cardiac output in rats with myocardial hypertrophy (Li G. et al., 2015). Pharmacological studies have shown that Fuling has a diuretic effect. It has been reported that it may be due to the Fuling-induced activation of the Na^+^ K^+^ ATPase on the cell membrane (Yang, 2012).

#### 6.2.4 Aurantii Fructus Immaturus (Zhishi, *Citrus aurantium* L.)

Zhishi is the dried young fruit of *Citrus aurantium* L. and its cultivated variety or sweet orange (*Citrus sinensis* Osbeck). It tastes bitter, spicy, and sour. It is slightly cold in nature, and it can dissolve Qi, dissipate over-accumulation, dissolve phlegm, and remove nodules. Zhishi is mainly used to treat accumulation-related internal stagnation, swelling pain, phlegm-induced stagnation, chest obstruction, chest knot, and so on. Zhishi is a common prescription as a mild soothing agent of the chest and is frequently prescribed for the treatment of coronary heart diseases in the clinic (Lv, 2017).

Studies have shown that the main active components of Zhishi are flavonoids, volatile oil, and a number of alkaloids. Flavonoids are the most abundant components in Zhishi, including dihydroflavones and polymethoxyflavones. Alkaloids are the main components of Zhishi to strengthen the heart and improve blood pressure, mainly including synephrine, n-methyltyramine, quinoline, and narcotine and norepinephrine (Yang, 2007; Zhang et al., 2009; Han et al., 2010). Several flavonoids in Zhishi can improve gastric emptying and small intestinal propulsion in rats with functional dyspepsia, and the effect of hesperidin on gastric emptying and small intestinal propulsion may be related to the increase in motilin secretion (Huang et al., 2012). Flavonoids in Zhishi can improve the symptoms of gastric ulcer induced by indomethacin in rats, mainly through the expression of gastric cyclooxygenase-2 (COX-2) (Hamdan et al., 2014). Research also showed that flavonoids from Zhishi inhibited adipogenesis through the Akt signaling pathway in 3T3-L1 cells (Ji et al., 2003; Jiao et al., 2007; Kim et al., 2012). It is reported that Zhishi can enhance myocardial contractility, increase cardiac output, improve heart pumping function, increase coronary flow, which would reduce myocardial oxygen consumption, and improve myocardial metabolism (Xu et al., 2009).

#### 6.2.5 Dichroae Radix (Changshan, *Dichroa febrifuga* Lour.)

Changshan was first recorded in the “Shen Nong Ben Cao Jing.” It is the dried roots of *Dichroa febrifuga* Lour. Changshan is bitter and spicy in taste, and it has toxicity when used inappropriately. It promotes the health of the lungs, the liver, and the heart. It is often used in the treatment of malaria.

The main active components in Changshan are quinazolone alkaloids. Its total alkaloid content is about 0.1%. The main alkaloids are changshanine, anomaline, neochangshanine, and berberine. Pharmacological studies have shown that Changshan has antimalarial, antipyretic, and antineoplastic effects. Intravenous injection of Changshan alkali into anesthetized dogs can reduce the amplitude of cardiac contraction and increase the volume of the spleen and the kidney (Zhang and Huang, 1956). Changrolin is an antiarrhythmic drug that is a chemical derivative of Changshan B. Changrolin has a negative inotropic effect, which can reduce the automaticity of the papillary muscle, prolong the functional refractory period, and reduce the excitability and the APA. Changrolin’s effects have been observed to be similar to those of quinidine (Yu et al., 1997). Changrolin inhibited delayed rectified K^+^ currents (I_K_), and it also induced a concentration-dependent inhibition of sodium currents (I_Na_) (Chen et al., 2010).

Prof. Ding used Qinghao and Changshan together to treat arrhythmia. The combination of the two herbs has a significant effect of clearing away heat and resolving phlegm, generating superior therapeutic effects. The cardiovascular cases treated by Prof. Ding in the last 10 years were further analyzed. A total of 2,850 cases of tachyarrhythmia such as premature cardiac contraction were treated. 90% of the patients were accompanied with PHHD symptoms, and they were treated with Qinghao, Changshan, and other herbs (Fan et al., 2020). Studies have shown that Qinghao and Changshan can significantly reduce the incidence of arrhythmia induced by pituitrin and coronary artery ligation and shorten the duration and frequency of arrhythmia. The combination of Qinghao and Changshan has protective effect on arrhythmia induced by aconitine, barium chloride, and chloroform and has a protective and therapeutic effect on arrhythmia caused by acute myocardial ischemia induced by coronary artery ligation in dogs (Jiao, 2006; Liu, 2007).

#### 6.2.6 Nelumbinis Plumula (Lianzixin, *Nelumbo nucifera* Gaertn.)

Nelumbinis Plumula is the dried spire and radicle of the mature seeds of *Nelumbo nucifera* Gaertn., a plant of the Nymphaeaceae family (Zhao, 2018). Lianzixin tastes bitter, and it is cold in nature. It could regulate the heart, lungs, and kidney function. Lianzixin has been used to treat insomnia, anxiety, spermatorrhea, thirst, swelling, and pain of the eyes (Song et al., 1995).

Research into the chemical compositions of Lianzixin revealed that the main components are alkaloids and flavonoids, which have shown pharmacological effects including antitumor, cardiovascular protection, antioxidation, inhibiting liver fibrosis, lowering blood sugar, bacteriostasis, and anti-inflammation (Dong et al., 2012; He and Yu, 2012; Liao and Lin, 2012). Animal experiments have shown that phenolic alkaloids in Lianzixin can shorten the recovery time of the sinus rhythm and reduce the mortality rate and can reduce the incidence of ventricular fibrillation in mice induced by chloroform and delay the occurrence time of ventricular fibrillation in mice (Zeng et al., 2007). Studies have shown that liensinine and neferine, which are extracts of Lianzixin, can inhibit the transmembrane transport of Na^+^, Ca^2+^, and K^+^ and produce synergistic antiarrhythmic effect (Wang et al., 1992; Dong et al., 2012). Besides, isoliensinine may antagonize stretch-induced arrhythmia by blocking stretch-activated ion channels (Chen et al., 2006).

In addition, silica gel column chromatography, ODS column chromatography, and RP- HPLC were used to study the chemical constituents of the ethyl acetate fraction of the 80% ethanol extract of Lianzixin. Four compounds were isolated, and their structures were identified by physicochemical properties and modern spectroscopy: (1*R*,1′*R*) Neferine *N*-Oxide, (1*R*,1′*R*) Neferine, liensinine, and isoliensinine. Among them, (1R, 1′r) neferine N-oxide is a new compound with no pharmacological properties being reported in the literature (Zhang, 2017a).

#### 6.2.7 Sophorae Flavescentis Radix (Kushen, *Sophora flavescens* Ait.)

Kushen is the dry root of *Sophora flavescens* Ait., a plant of the Leguminous family (Zhao et al., 2015). Kushen has a bitter taste, and its thermal properties belong to the cold category. Kushen has effects on the heart, liver, stomach, large intestine, and bladder meridians and could dry dampness, promote urination, kill parasites, and stop itching. It could treat diseases including phlegm-heat diseases, jaundice, and itching skin. Furthermore, “Ben Cao Xin Bian” recorded that “Kushen is used to eliminate sudden heartache” (Chen, 2018). The main bioactive components of Kushen are alkaloids and flavonoids.

A large number of literatures have shown that Kushen alkaloid (matrine) compounds have antitumor, anti-atherosclerosis, antivirus, immunosuppressive, hepatoprotective and cholagogic, anti-parasitic, and other pharmacological effects (Chen et al., 2016). Matrine reduces the amplitude of the action potential under arrhythmic conditions, implying the sodium channel blockade effect (Li et al., 2012), and weakens the effect of the I_KM3_ current (Sui et al., 2008) and has a significant effect on arrhythmia induced by low calcium and low magnesium (Zhang et al., 2008). The blocking of I_kr_ by matrine may be one of the mechanisms of prolonging the effective refractory period, reducing the incidence of the ectopic rhythm and treating arrhythmia (Huang and Liu, 2008). Besides, oxymatrine has some effects on arrhythmia caused by abnormal electrophysiology of the slow-response autonomic cells in the left ventricular outflow tract induced by ischemia and hypoxia (Li X. et al., 2015). Animal experiments have confirmed that oxymatrine can effectively prolong the QT interval and the effective refractory period, increase the excitability threshold of the myocardial diastolic phase, promote the decrease in myocardial cell autonomy, and trigger activity (Wang and Liu, 2011; Chen et al., 2018).

#### 6.2.8 Artemisiae Annuae Herba (Qinghao, *Artemisia annua* L.)

Qinghao is the dried aerial part of *Artemisia annua* L. It has a bitter and spicy taste and is cold in nature. It promotes the health of the liver and the gall bladder. Qinghao has effects on the liver and gall bladder meridians and could treat diseases including indigestion, malaria, constipation, gastrointestinal disorders, distention, and jaundice.

The main chemical constituents of Qinghao are sesquiterpenoids, dahlias, coumarins, phenylpropionic acid, and volatile oils (Huizhen et al., 1998). Studies have shown that Qinghao can significantly inhibit arrhythmia in rats induced by coronary ligation, electrical stimulation, or aconitine. It was found that artemisinin could inhibit the release of intracellular Ca^2+^ by interfering with outward rectifying K^+^ current in ventricular myocytes (Qiao et al., 2007). Artemisinin can significantly prolong the atrial effective refractory period and shorten the duration of atrial fibrillation in rats. It is speculated that artemisinin can upregulate the expression level of the Cav1.2 calcium channel and downregulate the expression levels of CaMK Ⅱ, resulting in the inhibition of the p-Ry R2 level, which has a therapeutic effect on atrial fibrillation in rats (Wang and Pei, 2020). The effects of artemisinin and amiodarone on the QT interval and the QRS interval in rats were observed by using the rat arrhythmia model induced by barium chloride. The results showed that the effect of artemisinin (20.0 mg/kg) on the shortening QT interval was better than that of amiodarone and with fewer side effects (Li et al., 2020). Artemisinin can significantly improve the cardiac systolic function of diabetic cardiomyopathy by improving myocardial fibrosis and improve cardiac diastolic function to some extent (Li et al., 2016), and artemisinin can inhibit left ventricular hypertrophy and improve the cardiac function of adult rats after coarctation of the aorta (Reid et al., 2016). Qinghao can also protect the heart from myocardial ischemia induced by pituitrin in rats and accelerate the heart rate. In addition, Qinghao has anti-malaria, anti-schistosomiasis, anti-asthmatic, anti-systemic lupus erythematosus, and antitumor effects (Shi, 2015).

#### 6.2.9 Ginseng Radix et Rhizoma (Ginseng, *Panax ginseng* C. A. Mey)

Ginseng is a perennial herb, a plant of the Araliaceae family (Li Q. et al., 2019). Ginseng has a sweet, slightly bitter taste and is mildly warm in nature. It promotes the health of the digestive system, lungs, and heart. It can be used to treat fatigue, shortness of breath, and chronic cough due to the deficiency of lungs and calm the mind (Wu, 2016).

Ginsenoside and Ginseng polysaccharides are the main chemical constituents in Ginseng. 3–5% of Ginseng roots and rhizomes is ginsenoside (Yang, 2016). Modern studies have shown that ginsenosides can inhibit arrhythmia by affecting potassium and calcium channels and protecting myocardial cells from injury caused by ischemia reperfusion or chemical reagents (Chen et al., 2015).

It was found that ginsenoside Rb1 significantly inhibited the L-type calcium current and the transient outward potassium current in ventricular myocytes (Pei et al., 2011). Ginsenoside Re can inhibit the voltage-dependent sodium current and the transient outward potassium current in ventricular myocytes (Yong et al., 2013). Ginsenoside Rg1 can promote the formation of coronary collateral vessels in the ischemic myocardium of rats, which may be related to the promotion of vascular endothelial growth factor expression and the recovery of ischemic heart function (Zhang et al., 2013). Ginsenoside Rg3 can slow down the apoptosis of rat cardiomyocytes and protect the myocardium (Wang Y. et al., 2015). In addition, Ginsenoside Rb can inhibit ventricular remodeling, improve myocardial ischemia, and protect the myocardium (You and Tian, 2021). Upon combined use of ginsenoside Re (20 μmol/L) and ginsenoside Rg1 (80 μmol/L), the inhibitory effect of the L-type calcium channel was stronger than those of ginsenoside Re and Rg1 alone (Han et al., 2019). Therefore, it is speculated that the antiarrhythmic effect of ginseng may be a combination of several effective components.

#### 6.2.10 Ophiopogonis Radix (Maidong, *Ophiopogon japonicus* (L. f) Ker Gawl.)

Maidong is the dried tuberous root of the perennial evergreen herb *Ophiopogon japonicus*. Maidong is sweet and bitter in taste and cold in nature. It has effects on the heart, lungs, and stomach, generates fluids, and moistens lungs and relieves cough. It can be used for hemoptysis, thirst, dry mouth, irritability, and constipation. “Shen Nong Ben Cao Jing” listed Maidong as the top grade of nourishing moistening lungs and stated that taking Maidong over the long term would lead to retaining youthful vitality (Wu, 2016; Zhang, 2017b).

The main active components of Maidong are steroidal saponins and hyper-isobrass (Bai, 2014). Pharmacological studies have shown that Maidong has anti-cardiovascular and cerebrovascular diseases, anti-aging, anticancer, and anti-inflammatory properties as well as regulating the functions of the immune system. Maidong may have antiarrhythmic effects by improving myocardial blood supply. Maidong saponins can reduce the excitability and automaticity of the right atrium muscles and prolong the refractory period of the left atrium (Yu et al., 2014). Maidong polysaccharide MDG-1 can enhance the tolerance of myocardial ischemia and hypoxia, increase coronary blood flow, protect ischemic myocardial cells, and promote the proliferation and differentiation of endothelial progenitor cells in rats with myocardial ischemia-reperfusion. It can also reduce the content of ischemia-modified albumin in blood, repair the ischemic myocardium to the greatest extent, and restore cardiac function (Xin et al., 2011).

#### 6.2.11 Nardostachyos Radix et Rhizoma (Gancao, *Glycyrrhiza uralensis* Fisch)

Gancao is the dried root and rhizome of the leguminous plant *Glycyrrhiza uralensis*, *Glycyrrhiza inflata*, or *Glycyrrhiza glabra*. Gancao is sweet in taste and mild in nature. It has effects on the heart, lungs, and digestive system and could benefit Qi. It moistens lungs and stops coughing, moderates spasms, stops pain, and clears heat. It also harmonizes the harsh characteristics of other herbs and eliminates various toxic actions. It could be used to treat fatigue, loose stools, irregular or intermittent pulse, shortness of breath, painful spasms of the abdomen or legs, carbuncles, sores, and soreness of the throat. “Shang Han Lun” recorded a famous prescription for the treatment of palpitation and irregular heartbeats, and the prescription takes Gancao as the principal herb (Li and Liu, 1985).

The main components of Gancao are glycyrrhizic acid and glycyrrhizin (Chen et al., 2000). Modern studies have shown that Gancao can inhibit arrhythmias caused by aconitine and ouabain. It can also shorten the time of arrhythmias induced by BaCl_2_ in rats and significantly slow down heart rates, and its effects are dose dependent. It can protect the myocardium and has obvious anti–myocardial ischemia activities (Pan, 2004; Zhang et al., 2015). Gancao can also reduce ectopic pacing and improve ECG conduction (Xu and Zhang, 1997).

In addition, since ancient times, Gancao has had the reputation of “detoxifying hundreds of herbs.” Chinese doctors often added Gancao to decoctions to reduce or eliminate its toxicity. Modern clinical application of Gancao can antagonize the toxicity of some chemical drugs and can also treat heavy metal poisoning, such as arsenic (Li P. et al., 2019). Pharmacological studies show that the material basis of reducing toxicity of Gancao is glycyrrhizin and glycyrrhetinic acid (Fujisawa et al., 2000; Jung et al., 2017; Wang and Han, 2018). This is one of the reasons why the combination of multiple herbs can not only enhance the curative effect but also reduce the side effects.

### 6.3 The Forming Principles of Xin Su Ning

The XSN capsule is tailored toward the pathogenesis of the “heated phlegm with heart disturbance causing blockade of the heart meridian” type of arrhythmia. Modified based on the ancient Huanglian Wen Dan Decoction, XSN is formulated with the aforementioned herbs: Huanglian, Banxia, Fuling, Zhishi, Changshan, Lianzixin, Kushen, Qinghao, Ginseng, Maidong, and Gancao. Its effects include clearing the heat and dissolving phlegm, replenishing Qi, and relieving palpitation.

The principle of “Emperor–Minister–Adjuvant–Courier” elaborates the status of each herb in the formulation (Kaptchuk, 2014). Emperors are the herbs in the formula used to treat the main symptoms of the disease. They reflect the principal direction of the formula, and they are indispensable. Huanglian and Banxia are the emperors of XSN, whose principal effects are resolving phlegm and clearing the heat of the heart, respectively. Besides, the cold nature of Huanglian is neutralized by the warm nature of Banxia.

Ministers are herbs which enhance the actions of the “Emperor” to treat the main syndrome or disease. Lianzixin, Qinghao, and Changshan are ministers of XSN. They help Huanglian and Banxia to resolve phlegm and clear the heat of the heart (Yuan, 2000). They both enter the heart meridian and could clear away the heart disturbances and calm the mind. When used together, they will remove phlegm and clear heat and relieve irritation.

Adjuvants are the herbs that are used to treat subordinated and accompanying symptoms and reduce potential side effects of Emperors or Ministers. Fuling, Zhishi, and Kushen are the adjuvants to regulate Qi and dissipate phlegm (Li, 2005). Fuling is mild in nature. It promotes the health of the heart. Zhishi is slightly cold in nature and promotes the health of the digestion. It could help digestion, dissolve phlegm, and remove abnormal nodules. Kushen has the effect of dissolving phlegm and relieving uneasiness of the mind and tranquilizing the body at the same time. It could strengthen the digestion, help to resolve phlegm, and clear heat.

Couriers are herbs that help to coordinate the drug actions in the formulation and make them work better together and also reduce side effects. Ginseng, Maidong, and Gancao are couriers in the decoction. Gancao can tonify Qi, harmonize the herbs, and reduce the side effect as well. Ginseng is mild and slightly warm in nature and could strengthen the organs, tonify Qi, and calm the mind. Maidong is slightly cold in nature. It affects the heart, lungs, and digestive system. The combination of Ginseng and Maidong is used like Sheng Mai Decoction to relieve palpitation and shortness of breath and calm the mind.

Overall, the whole prescription nourishes the body and balances the Qi and the blood. It aims to harmonize yin and yang, dissolve the phlegm-heat, relieve mental stress, resist fear, and stop palpitation.

## 7 The Limitations of the Available Basic and Clinical Studies of XSN and Possible Further Studies

The fundamental difference between new drug discovery studies and the pharmacological studies of TCM is the former being initiated in scientific research laboratories and the latter being based on long-time clinical use in patients. Therefore, how to rigorously evaluate the clinical efficacy of TCM has been the most important question in this field of study. The cellular electrophysiological study is the conventional method used in antiarrhythmic drug discovery studies, and the data generated form the base for antiarrhythmic drug classifications. Hence, the available data on cellular electrophysiological property of XSN were reviewed in this article. However, in order to fully understand the action mechanisms of the multicomponent XSN and its clinical efficacy in comparison with other antiarrhythmic drugs, there are more basic and clinical studies that need to be carried out:

1. The effect of XSN in cardiac arrhythmic models, including various diseases’ model-induced arrhythmias.
2. Clinical studies with longer periods and more patients to compare the differences between XSN and other antiarrhythmics in therapeutic and toxicological profiles.
3. The pharmacological studies of all the active components of XSN.
4. Bioinformatic studies of XSN’s clinical efficacy.
5. The quality standard and control studies of XSN.

## 8 Conclusion

XSN as a clinically effective antiarrhythmic TCM displays the characteristics of class I and III antiarrhythmics, which was confirmed using conventional cellular electrophysiological research methods that have been used for discovering antiarrhythmic drugs for the last few decades. The unique property that XSN possesses is its safety profile in comparison with other antiarrhythmic drugs.

The multicomponent nature of XSN has made it multitargeting, which allows XSN to exert its cardioprotective actions while regulating the ion channels to suppress cardiac arrhythmias.

XSN is the first TCM approved in China for the treatment of PHHD arrhythmia according to Chinese medicine theories. The 11 herbs that formulated XSN display various pharmacological properties; following TCM theory, they were mixed with particular proportions of each herb that formulated XSN. The safety profile of XSN gives it the potential of further research and development.

We hope that the advances in how XSN was studied may offer useful guidance on how other TCM could be studied with respect to the integrity of the TCM formulas.

### Comment

All the classical books of traditional Chinese medicine cited in this article were written in ancient times and then compiled by modern scholars. The year in the reference is the time when the book was published by modern scholars.

## Abbreviations

AAD, anti-arrhythmic drugs; AECG, abdominal electrocardiograph; ALT, alanine aminotransferase; AP, action potential; APD, action potential duration; AST, aspartate aminotransferase; CI, confidence interval; ECG, electrocardiogram; ER, effective response; FAS, full analysis set; HPLC, high-performance liquid chromatography; HR, heart rate; I/R, ischemia-reperfusion; ISL, isoliquirigenin; LVDP, left ventricular diastolic pressure; MW, molecular weight; ODS chromatography, octadecyl silane column chromatography; PHHD, phlegm-heat heart-disturbed syndrome; PVC, premature ventricular contraction; RP-HPLC, reversed-phase high performance liquid chromatography; RPP, rate-pressure product; Scr, serum creatinine; SER, significantly effective response; TCM, traditional Chinese medicine; TER, total effective response; XSN, Xin Su Ning.
